# Semantic Analysis of Posttranslational Modification of Proteins Accumulated in Thyroid Cancer Cells Exposed to Simulated Microgravity

**DOI:** 10.3390/ijms19082257

**Published:** 2018-08-01

**Authors:** Johann Bauer, Markus Wehland, Manfred Infanger, Daniela Grimm, Erich Gombocz

**Affiliations:** 1 Max-Planck-Institute for Biochemistry, 82152 Martinsried, Germany; jbauer@biochem.mpg.de; 2 Clinic and Policlinic for Plastic, Aesthetic and Hand Surgery, Otto-von-Guericke-University, 39120 Magdeburg, Germany; markus.wehland@med.ovgu.de (M.W.); manfred.infanger@med.ovgu.de (M.I.); 3 Department of Biomedicine, Aarhus University, Aarhus C 8000, Denmark; 4 Melissa Informatics, 2550 Ninth Street, Suite 114, Berkeley, CA, USA; egombocz@ix.netcom.com

**Keywords:** mass spectrometry, Knowledge Explorer, Reactome, pathway analysis, random positioning machine, semantic network, SPARQL reasoning

## Abstract

When monolayers of tissue cancer cells of various origins are exposed to real or simulated microgravity, many cells leave the monolayer and assemble to three-dimensional (3D) aggregates (spheroids). In order to define the cellular machinery leading to this change in growth behavior of FTC-133 human thyroid cancer cells and MCF-7 breast cancer cells, we recently performed proteome analyses on these cell lines and determined the proteins’ accumulation in monolayer cells grown under 1*g*-conditions as well as in the cells of spheroids assembled under simulated microgravity during three and 14 days, respectively. At that time, an influence of the increment or decrement of some of the more than 5000 proteins detected in each cell line was investigated. In this study, we focused on posttranslational modifications (PTMs) of proteins. For this purpose, we selected candidates from the list of the proteins detected in the two preceding proteome analyses, which showed significant accumulation in spheroid cells as compared to 1*g* monolayer cells. Then we searched for those PTMs of the selected proteins, which according to the literature have already been determined experimentally. Using the Semantic Protocol and RDF Query Language (SPARQL), various databases were examined. Most efficient was the search in the latest version of the dbPTM database. In total, we found 72 different classes of PTMs comprising mainly phosphorylation, glycosylation, ubiquitination and acetylation. Most interestingly, in 35 of the 69 proteins, N6 residues of lysine are modifiable.

## 1. Introduction

The occurrence of thyroid cancer is rising, especially among women. Worldwide, this tumor has become the seventh most common cancer among women [1,2]. If the progress of this cancer is driven by differentiated epithelial cells, it can frequently be cured [3]. Poorly differentiated or anaplastic types of thyroid cancer, however, have an unfavorable prognosis and current therapy options are limited [4,5]. Therefore, new strategies have been investigated in order to find novel targets for therapy of this kind of tumor [6] and to prevent metastasis [7]. 

For several years we have investigated the behavior of malignant thyroid cells, putting emphasis on cellular differentiation and on their migration [8]. In this context, we exposed human follicular thyroid cancer cells to simulated and real microgravity (µ*g*) in vitro [9]. Under this condition, a part of the cells leaves the two-dimensional (2D) monolayers. Normally, thyroid cancer cells grow adherently in a 2D layer in vitro when they are seeded in culture flasks and incubated in an incubator on Earth at 1*g* normal laboratory conditions. The cells leaving monolayers assemble scaffold-free into three-dimensional (3D) aggregates (so-called spheroids) and continue to grow there [10]. This change of phenotype happens during spaceflights, as well as during cell cultivation on devices preventing sedimentation by annulling or randomization of the gravity vector [10,11,12,13,14]. It is accompanied by structural changes of the cytoskeleton as well as by alterations of the mRNA expression pattern and the protein production [15,16,17] and appears to mimic metastasis-like scaffold-supported cell migration [18]. 

Mass spectrometry is a powerful method to analyze the protein production of human cells [19]. It currently is applied in many fields including space research [20,21,22]. Recently, we performed deep proteome analyses on thyroid cancer cells unveiling about 5900 proteins, and on MCF-7 breast cancer cells for comparison, aiming to shed light on the molecular machinery, which enables the cells to move from one kind of growth (monolayer) to another one (spheroid) [17,23]. The studies revealed a number of proteins, whose accumulations were significantly different, when they were found either in monolayer cells of normal 1*g* cultures or in spheroids formed under simulated microgravity. Taken together they suggested that there is a relationship between the increment and decrement of distinct proteins and the kind of growth of the cells [17,23]. Furthermore, we observed phosphorylation of profilin-2 and de-phosphorylation of extracellular signal–regulated kinases (ERKs) 1/2 on thyroid cells exposed to microgravity [24,25] and recognized that genes mostly up- and down-regulated during a ten-day space mission coded for enzymes involved in posttranslational modifications (PTM) of target proteins [26]. The observations are in accordance with publications of other researchers [27,28]. Hence, it is probable that a protein’s activation status determined by PTM, such as phosphorylation, glycosylation, ubiquitination and others [29,30], is also important for the cellular behavior under µ*g*.

Consequently, we intend to gain comprehensive information about PTMs, which occur in thyroid cells forming a spheroid. We expect various PTMs to occur in a tremendous number of proteins [31,32], which appear to be involved in the cells leaving a monolayer and joining a 3D aggregate. Hence, we assume that a high number of experiments will be required until a reasonable overview will be obtained. In order to keep this number as low as possible, in this study we try to find PTMs that, according to the literature, have already been observed on proteins involved in spheroid formation. 

PTMs already noticed by experiments are stored in various public repositories including the newly extended comprehensive dbPTM database [33]. Using Semantic Protocol and RDF Query Language (SPARQL) endpoints and a semantic data model allows the retrieval and handling of data stored in Resource Description Framework (RDF) format for further interlinking of various databases and enrichment of information through inference queries. In order to accomplish this, we applied the Sentient Knowledge Explorer (KE), semantic retrieval software that enables mapping, aligning and merging of information from several relevant resources [34,35], to establish the most comprehensive evidence possible. 

## 2. Results and Discussion

### 2.1. Selection of Proteins from a Set of Data Obtained in a Preceding Mass Spectrometry (MS) Analysis

69 proteins of interest (Table 1) were selected from a list of 5989 human thyroid proteins identified and quantified in a recent proteome study on FTC-133 thyroid cancer cells, which grew in a monolayer under normal 1*g* laboratory conditions or within spheroids exposed to a random positioning machine (RPM) simulating microgravity during three days of incubation [17]. These proteins were of interest because they were found in spheroid cells but not in control monolayer cells, or were found in spheroid cells at a 1.8-fold higher accumulation than in control cells. In addition, they also were detected by a second independent proteome analysis on MCF-7 breast cancer cells, which grew in a monolayer under normal 1*g* laboratory conditions or within spheroids exposed to an RPM for 14 days [23]. Their up-regulation in spheroid cells as compared to control cells was similar to those of the corresponding FTC-133 cells (Table 1). The selected 69 proteins comprised 1.2% of the total proteins detected in the FTC-133 spheroid cells. Since each of these proteins was detected in at least four samples being accumulated in spheroids of FTC-133 cells after three days and in MCF-7 spheroids after 14 days, they were considered to be candidates for triggering a transition from a 2D to a 3D kind of growth. In order to prove this hypothesis, they were further analyzed applying in silico methods.

### 2.2. Characterization of the Selected 69 Proteins According to Localization and Interaction

**Figure 1:**
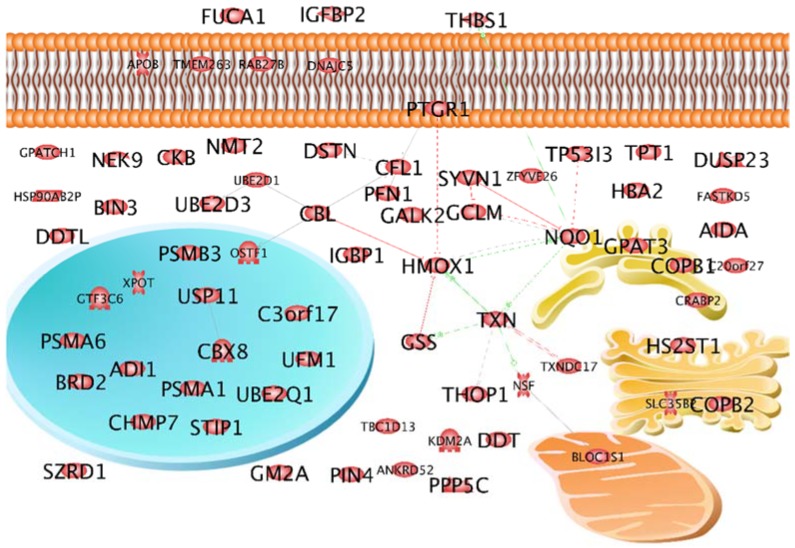
Localization of the selected proteins and their interactions on gene and protein levels. Blue circle indicates nucleus, yellow figure indicates endoplasmatic reticulum, orange shows the mitochondrion and ocher the Golgi apparatus. Solid lines indicate binding, solid arrows indicate regulation by direct interaction, dashed arrows indicate indirect regulation via other cellular components. Green arrows indicate up-regulation, red arrows indicate down-regulation.

In a first approach, the cellular location of the selected proteins was studied using the Elsevier Pathway Studio v11 (Elsevier, Amsterdam, the Netherlands). Figure 1 shows that the majority of these proteins are located within the cytoplasm. But others are active within the nucleus, the mitochondria, the endoplasmic reticulum ER, the membrane and the extracellular matrix. At that point, it was of interest to see whether the selected proteins represent single independent hits or are members of a network. The Pathway Studio analysis revealed that 20 of these proteins form a network of interaction either on a gene level or on a protein level. Central components of the network were heme oxygenase 1 (*HMOX1*), thioredoxin (*TXN*) and NAD(P)H dehydrogenase [quinone] 1 (*NQO1*) (Figure 1). HMOX1 and NQO1 are both regulated by the transcription factor Nrf2 [36]. HMOX1 was of special interest because it had already shown up in an earlier study and was considered as important for spheroid formation [37]. In addition to the interactions shown in Figure 1, it has relationships to nuclear factor kappa B [25], to connective tissue growth factor, caveolin 1, and to intercellular adhesion molecule 1. All these proteins were found in earlier studies and were considered as key proteins of spheroid formation [24,38,39]. 

### 2.3. Creation of a Semantic Knowledge Base on PTM Modification

**Figure 2:**
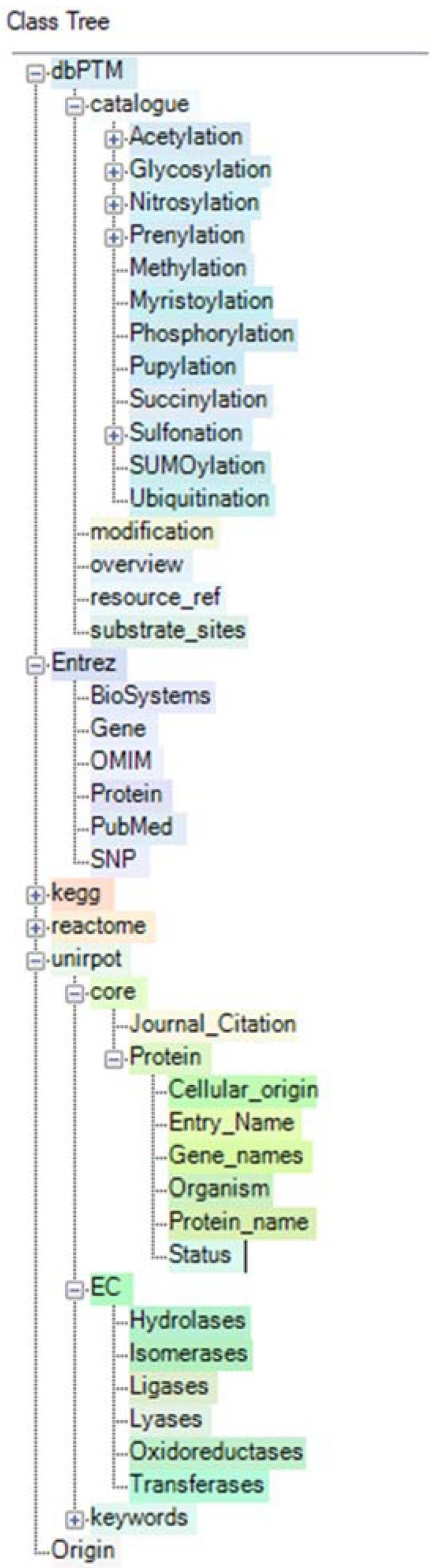
Databases, with their partially extended ontological class trees, whose content was searched via Semantic Protocol and RDF Query Language (SPARQL) using Knowledge Explorer (KE) [34,40]. Retrieved results were imported, harmonized and mapped to this ontology to create a Semantic Knowledge Base.

Regarding the 69 proteins shown in Table 1, which were selected due to their differences of accumulation in monolayer cells exposed to gravity as compared to spheroids cultured under microgravity, we were not only interested in cellular localization and interaction. We also focused on their posttranslational modifications, which generally have a great influence on cellular behavior [29,30]. Since a great number of PTMs could theoretically be considered for the selected 69 proteins, we retrieved only PTMs that, according to the literature, have already been verified for these proteins experimentally, and tried to set up a relationship to their function and their influence on the behavior of the cells. In order to find relevant literature and information about the proteins’ PTMs, we applied the KE, which enables searching of various databases for many PTMs and harmonization of the results. Databases searched included UniProt, Entrez, Reactome and dbPTM (Figure 2). These databases contain information supplementing each other, so that not only PTMs can be found but also their role within the concert of life.

Using KE, relevant information about each of the 69 proteins was collected from UniProt in a first step. For this purpose, a spreadsheet was created containing the names of the 69 proteins selected together with their gene names, UniProt accession numbers and experimental data, which indicated the individual cellular accumulation by LfQ scores (Table 1). 

**Figure 3:**
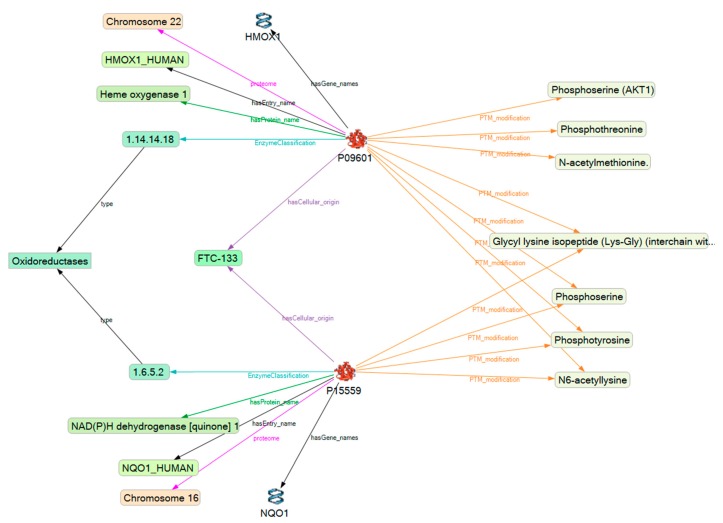
Uniprot derived starting network for heme oxygenase 1 and NAD(P)H dehydrogenase [quinone] 1. The network includes data about the proteins’ names and accession numbers, about enzyme classes, as well as about posttranslational modifications (PTMs) stored in UniProt [31]. Similar information was gathered for all the other proteins.

To build the initial Semantic Knowledge Base (SKB), the spreadsheet was imported into KE and mapping of the experimental data based on their protein accession numbers to UniProt’s SPARQL endpoint was applied [41]. A representative starting network for two selected proteins (Heme oxygenase 1 and NAD(P)H dehydrogenase [quinone] 1) from the 69 proteins of specific interest is shown in Figure 3.

**Figure 4:**
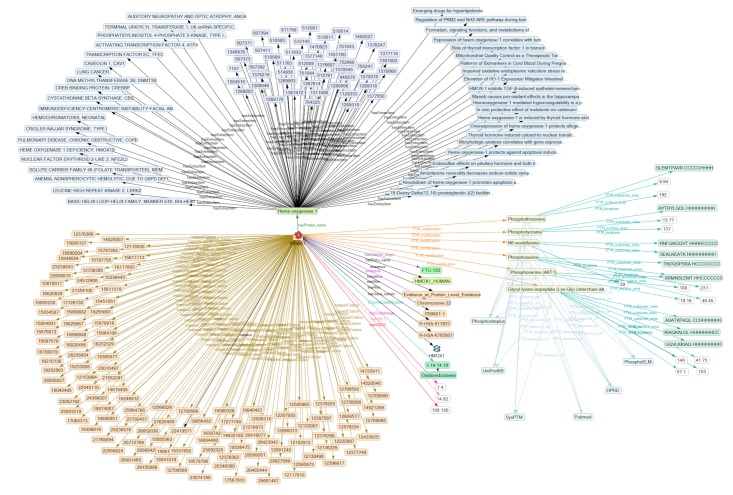
Results of a KE-mediated comprehensive search for information about HMOX-1 in UniProt, NLM-reference databases, and Reactome. The upper half shows NCBI Entrez data about HMOX1 restricted to thyroid (left to right: OMIM, Biosystems, Pubmed—violet blue); the lower left to center shows UniProt data (brown). On the right of the center line are classified PTMs (yellow-green) from dbPTM with their reference resources attached (light blue). (This is a high-resolution picture. A reader interested in detail may zoom in to amplify.).

In the next step, SPARQL queries were developed by selecting nodes from the canvas of the network graphs and applying filters via setting a certain element’s variable or by applying restrictions, such as ranges on numerical values, as described earlier [34,35]. They were translated to text queries by KE. After suitable formulation they could be used to access a number of databases. First, a query was formulated suitable to search selected U.S. National Library of Medicine (NLM) databases (Biosystems, Protein, Gene, Online Mendelian Inheritance in Man (OMIM), Single Nucleotide Polymorphisms (SNP)). Then the National Center for Biotechnology Information (NCBI) Entrez Application Programming Interface (API) Connector was used to import results from the SPARQL queries directly as RDF into the knowledge graph [42,43]. The information obtained augments the characterization of a selected protein and will help to relate PTMs to their biological processes and the signaling pathways involved. This is shown for heme oxygenase in regard to functional, genetic and biological aspects of human thyroid cells (Figure 4). The information was mainly included in literature references indicated by PubMed unique identifier (PMID) numbers.

Subsequently, further SPARQL queries were used to expand the SKB by importing the protein’s involvement in signaling pathways from Reactome [44], after cross reference with pathway information stored in the Kyoto Encyclopedia of Genes and Genomes (KEGG). In the lower part of Figure 4, the pathways found for heme oxygenase are indicated by black arrows to brown blocks. The numbers R-HAS-917937 and R-HAS-6785807 point to pathways of iron uptake and transportation, and of interleukin 4 signaling, respectively.

**Figure 5:**
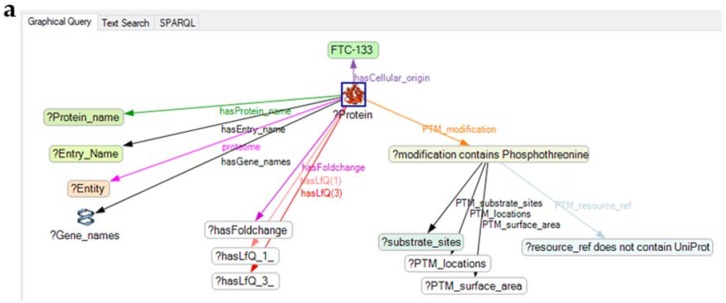

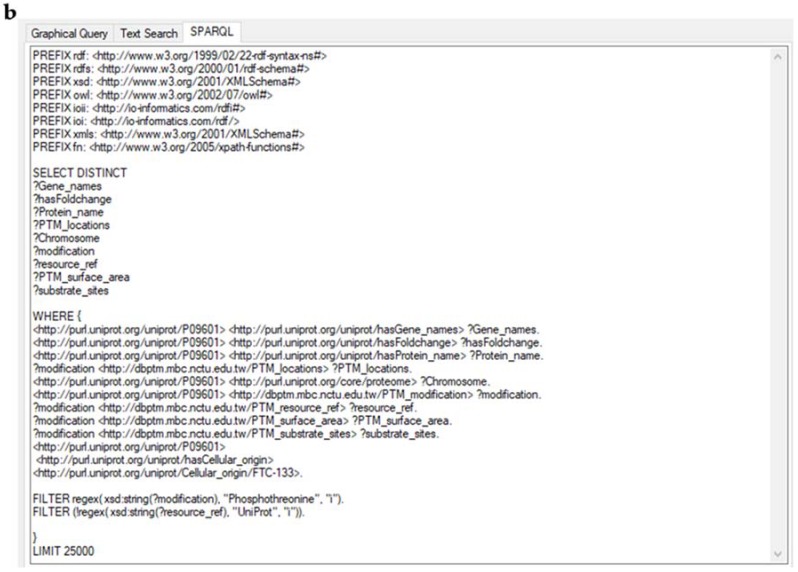
A typical graphical SPARQL query for searching for information about all proteins imported in the KE via a spreadsheet in databases of interest for the topic of a study (see also ref. [35]). The query shown directs KE to the dbPTM to search for substrate sites and location of modifications, as well as for the surface area covered by the modification (**a**). The graphical query is auto-translated into a SPARQL text query by KE and used for searches (**b**).

In a final step, PTM information about the proteins selected was retrieved [29]. For this purpose, a SPARQL query was formulated to search the database dbPTM [33]. The query contains information obtained from UniProt and directs the program to search PTMs in the dbPTM database for all proteins imported into KE via spreadsheet (Figure 5, Table 1). In the database, those fields were searched where information about the reference source, the type of a modification and the location with a short segment of the sequence modified can be found. In addition, the size of the area covered by the modification is available.

**Figure 6:**
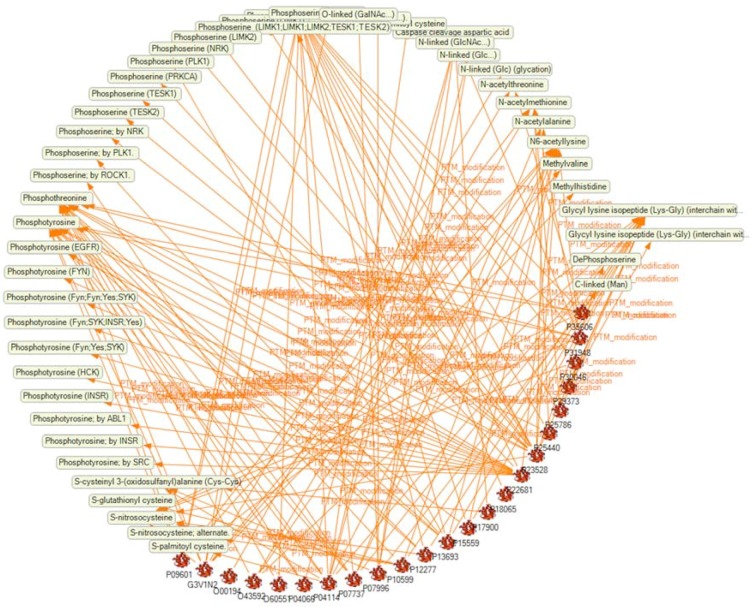
Overview of PTMs (yellow-green boxes) of 23 of the selected proteins (icons with accession numbers). The arrows indicate the relationship between a protein and a PTM.

Iterative use of queries with filters for the parameters of interest enabled us to accumulate comprehensive information about the PTMs of 69 proteins (see Supplementary Table S1). Figure 6 shows PTMs (yellow-green boxes) for 23 proteins (icons with accession numbers) selected from the total 69 proteins in order to keep a clear view on details. It can be seen that there are proteins with many PTMs and others with only a few or one. For example, cofilin-1, with the accession number P23528, shows arrows to a considerable number of PTMs, while the Ras-related protein, Rab-27B (O00194), shows only one arrow which links the icon to an *N*-acetylation of threonine. Taken together, it is obvious that the selected proteins are frequently modified by phosphorylation, ubiquitination and glycosylation. 

### 2.4. Analysis of the SKB on PTM Modification

The knowledge base created for 69 proteins is rather complex if it is represented as shown in Figure 6. Looking at such diagrams, recognizing detailed and useful information visually is difficult. Hence, the complexity was reduced by splitting the whole knowledge base into segments and visualizing and highlighting the results in individual graphs. For this purpose, graphical queries were applied to the entire network and their results highlighted. The aggregated query results are exported as spreadsheets to create final tables for all proteins involved in the study. Table 2 shows detailed data about five exemplary proteins. 

A summary of PTMs found for all of the 69 proteins is provided in the Supplementary Table S1, which shows the substrate sites already proved experimentally, but not sites theoretically modifiable, and indicates the accessible surface areas (ASA) covered by the modification. An evaluation of the table reveals 72 different classes of identified PTMs. In total, 406 classified PTMs have been counted as single count/protein for a specific class, even when multiple sites/protein have been affected. Of these, the most prominent classes were phosphorylation (48%; phosphoserine, phosphotyrosine, phosphothreonine) and lysine-N6 modification (18.9%), followed by modifications of other residual nitrogens (12.8%) or sulfurs (4%), and by glycin-lysin dipeptide coupling (3.4%). It should also be mentioned that 35 of the 69 proteins were modified on N6 groups of lysine. This observation is of great interest, because a tremendous down-regulation of the gene of the protein-lysine 6-oxidase (*LOX*) has been observed in FTC-133 cells during the Shenzhou-8/SimBox space mission [16,26]. The protein-lysine 6-oxidase catalyzes deamination of lysine residues [45]. 

## 3. Conclusions

Proteins change their influence on the life of a cell when differently accumulated within the cells and when modified after their translation. A comparative proteome analysis of thyroid cancer cells living within a monolayer under normal gravity or within spheroids under simulated µg, showed an up-regulation of 69 proteins detected in spheroids. Applying the KE, we searched in databases containing relevant literature and biological pathways. Most efficient was searching the dbPTM databases, which triplicated the result (Supplementary Table S1). Hence, we learnt details about the PTMs of each of the 69 proteins (Table 2, Supplementary Table S1). Their evaluation unveiled a high percentage of the 69 selected proteins with modifiable N6 lysine residues. Hence, the study shows a way to facilitate planning work on possible PTMs of proteins of cells actually changing their type of growth and offers an explanation of earlier findings regarding the *LOX* gene. In future, the method may complement even advanced methods of proteome analysis with PTM identification facilities [46].

## 4. Materials and Methods

### 4.1. Proteome Data Used

The proteins were obtained by mass spectrometry from FTC-133 human follicular thyroid carcinoma cells and from MCF-7 human breast adenocarcinoma cells according to protocols described in refs. [17,23]. Prior to analysis both types of cells had been grown either within a monolayer under normal 1*g* laboratory conditions or within spheroids exposed to an RPM [10]. After harvest, the cells were lysed and subjected to mass spectrometry, obeying the protocols described in refs. [17,23,47,48]. Finally, raw data from the mass spectrometer were processed using MaxQuant (May Planck Society, Munich, Germany) computational proteomics platform (version 1.5.2.22) [49] using the standard parameters. Relative protein concentration was performed using the LfQ algorithm (label free quantitation) as described in [50].

### 4.2. Pathway Analysis

To investigate and visualize the original localization and the mutual interactions of detected proteins, we entered relevant UniProt accession numbers in a Pathway Studio v.11 software (Elsevier Research Solutions, Amsterdam, The Netherlands) [17,23]. 

### 4.3. Application of the Knowledge Explorer

To create a semantic network, harmonize content from multiple resources, and allow for graphical querying and reasoning, experimental data were imported to establish an initial RDF knowledge base using Sentient Knowledge Explorer (Melissa Informatics, Berkeley, CA, USA—former IO Informatics) [34]. The workflow of the process to generate a knowledge base by iterative selective SPARQL queries with reasoning to those resources and importing their results into the semantic network is depicted in Figure 2. UniProt content was queried using its SPARQL endpoint to augment information on enzymes and reported protein functions [41]. The Entrez resources Gene, OMIM, Protein, PubMed and SNP were used via Knowledge Explorer’s NCBI Connector services to add content [42,43,51]. Parts of KEGG and Reactome were used to validate pathway information [44]. For classification of post translational modifications, the integrated resource for protein Post-Translational Modifications (dbPTM) was used [52]. The dbPTM is an integrated resource for protein post-translational modifications experimentally verified and annotating the potential PTMs for all UniProtKB protein entries [53]. dbPTM [33,53] is an aggregated protein-modification and protein-interaction database containing data from 7 sources (Uniprot, HPRB, PhosphoELM, Phosphositeplus, SysPTM [54], dbSNO [55], MeMo [56]), which categorize more than 80 classes of posttranslational modifications. It integrates experimentally verified PTMs from several databases and annotates the potential PTMs for all UniProtKB protein entries. Since the last update, dbPTM also provides disease association based on non-synonymous single nucleotide polymorphisms (nsSNPs). All PTMs experimentally confirmed for each protein were collected and denoted according to their PTM classification. The records were added to establish the final comprehensive SKB. 

## Figures and Tables

**Table 1:** List of 69 selected proteins that were imported into the Knowledge Explorer, with protein names, gene names, accession numbers and LfQs * obtained for proteins of FTC-133 cells, before it was used for searching UniProt. LfQ * values for MCF-7 cells are given for comparison.

Gene Name	Protein Name with Swiss Prot Accession Number	FTC-133		MCF-7	
		LfQ * [1*g*]	LfQ * [µ*g*]	LfQ * [1*g*]	LfQ * [µ*g*]
*RAB27B*	Ras-related protein Rab-27B (O00194)	0	1.11	7.24	22.81
*XPOT*	Exportin-T (O43592)	0	1.75	6.62	12.92
*NMT2*	Glycylpeptide *N*-tetradecanoyltransferase 2 (O60551)	0	0.31	0.39	0.72
*FUCA1*	Tissue alpha-l-fucosidase (P04066)	0	1.02	5.32	9.95
*THBS1*	Thrombospondin-1 (P07996)	0	3.96	3.89	11.1
*IGFBP2*	Insulin-like growth factor-binding protein 2 (P18065)	0	7.25	0.92	1.85
*CBL*	E3 ubiquitin-protein ligase CBL (P22681)	0	0.74	0.12	0.23
*UBE2D1*	Ubiquitin-conjugating enzyme E2 D1 (P51668)	0	0.49	0	1.14
*USP11*	Ubiquitin carboxyl-terminal hydrolase 11 (P51784)	0	0.98	0.27	0.56
*BLOC1S1*	Biogenesis of lysosome-related organelles complex 1 subunit 1 (P78537)	0	0.6	0.82	2.45
*GALK2*	*N*-acetylgalactosamine kinase (Q01415)	0	0.61	2.1	4.82
*TP53I3*	Quinone oxidoreductase PIG3 (Q53FA7)	0	1.52	0.41	0.77
*ZFYVE26*	Zinc finger FYVE domain-containing protein 26 (Q68DK2)	0	0.22	0.05	0.16
*C3orf17*	Uncharacterized protein C3orf17 (Q6NW34)	0	1.01	0.05	0.25
*FASTKD5*	FAST kinase domain-containing protein 5 (Q7L8L6)	0	1.02	0	0.46
*HS2ST1*	Heparan sulfate 2-O-sulfotransferase 1 (Q7LGA3)	0	3.13	0	0.37
*SZRD1*	SUZ domain-containing protein 1 (Q7Z422)	0	3.52	0	0.24
*UBE2Q1*	Ubiquitin-conjugating enzyme E2 Q1 (Q7Z7E8)	0	1.64	1.21	2.94
*SYVN1*	E3 ubiquitin-protein ligase synoviolin (Q86TM6)	0	0.69	1.09	4.39
*SLC35B2*	Adenosine 3-phospho 5-phosphosulfate transporter 1 (Q8TB61)	0	6.82	3.97	7.42
*NEK9*	Serine/threonine-protein kinase Nek9 (Q8TD19)	0	1.38	0.86	1.75
*CHMP7*	Charged multivesicular body protein 7 (Q8WUX9)	0	0.98	0.99	1.92
*GTF3C6*	General transcription factor 3C polypeptide 6 (Q969F1)	0	0.97	1.37	2.79
*AIDA*	Axin interactor. dorsalization-associated protein (Q96BJ3)	0	1.91	0	0.17
*GPATCH1*	G patch domain-containing protein 1 (Q9BRR8)	0	0.77	0	0.13
*ADI1*	1.2-dihydroxy-3-keto-5-methylthiopentene dioxygenase (Q9BV57)	0	1.32	16.08	29.15
*DUSP23*	Dual specificity protein phosphatase 23 (Q9BVJ7)	0	1.75	2.76	5.02
*C20orf27*	UPF0687 protein C20orf27 (Q9GZN8)	0	1.05	2.55	5.08
*DNAJC5*	DnaJ homolog subfamily C member 5 (Q9H3Z4)	0	1.84	7.88	14.6
*CBX8*	Chromobox protein homolog 8 (Q9HC52)	0	3.35	1.79	3.86
*BIN3*	Bridging integrator 3 (Q9NQY0)	0	1.34	0	0.27
*TBC1D13*	TBC1 domain family member 13 (Q9NVG8)	0	1.6	1.59	2.98
*IGBP1*	Immunoglobulin-binding protein 1 (P78318)	0.7	1.32	1.14	2.33
*ANKRD52*	Serine/threonine-protein phosphatase 6 regulatory ankyrin repeat subunit C (Q8NB46)	0.73	1.37	0	0.18
*THOP1*	Thimet oligopeptidase (P52888)	1.04	2.05	1.58	3.49
*AGPAT9*	Glycerol-3-phosphate acyltransferase 3 (Q53EU6)	1.25	2.46	0	0.13
*HBA2*	Hemoglobin subunit alpha (G3V1N2)	1.43	8.92	2.05	5.25
*UBE2D3*	Ubiquitin-conjugating enzyme E2 D3 (P61077)	1.6	4.05	0	2.26
*GM2A*	Ganglioside GM2 activator (P48637)	1.75	3.34	1.12	2.45
*KDM2A*	Lysine-specific demethylase 2A (Q9Y2K7)	1.87	5.59	1.47	4.13
*GSS*	Glutathione synthetase (P48637)	1.89	5.08	2.14	4.99
*TMEM263*	Transmembrane protein 263 (Q8WUH6)	2.29	4.19	3.66	9.68
*BRD2*	Bromodomain-containing protein 2 (P25440)	2.33	4.43	5.15	9.39
*GCLM*	Glutamate-cysteine ligase regulatory subunit (P48507)	3.91	9.31	0.30	1.93
*PPP5C*	Serine/threonine-protein phosphatase 5 (P53041)	3.99	9.22	3.96	7.77
*PTGR1*	Prostaglandin reductase 1 (Q14914)	5.25	11.92	1.21	5.62
*PIN4*	Peptidyl-prolyl cis-trans isomerase NIMA-interacting 4 (Q9Y237)	5.59	12.75	5.64	10.88
*TXNDC17*	Thioredoxin domain-containing protein 17 (Q9BRA2)	5.88	14.58	14.38	52.39
*PSMB3*	Proteasome subunit beta type-3 (P49720)	5.92	10.72	1.05	2.5
*HSP90AB2P*	Putative heat shock protein HSP 90-beta 2 (Q58FF8)	6.19	13.49	9.79	36.46
*DDT*	D-dopachrome decarboxylase (P30046)	6.46	13.31	1.35	5.21
*OSTF1*	Osteoclast-stimulating factor 1 (Q92882)	7.47	19.57	2.97	7.56
*UFM1*	Ubiquitin-fold modifier 1 (P61960)	7.89	15.94	12.82	25.55
*APOB*	Apolipoprotein B-100 (P04114)	8.67	49.51	0.10	10.18
*CRABP2*	Cellular retinoic acid-binding protein 2 (P29373)	8.88	17.32	13.46	65.46
*PSMA1*	Proteasome subunit alpha type-1 (P25786)	13.84	27.82	5.4	10.7
*HMOX1*	Heme oxygenase 1 (P09601)	14.62	108.19	0.58	1.39
*COPB1*	Coatomer subunit beta (P53618)	16.3	37	83.81	122.1
*CKB*	Creatine kinase B-type (P12277)	21.21	40.3	0.72	1.35
*NQO1*	NAD(P)H dehydrogenase [quinone] 1 (P15559)	22.76	56.44	65.66	183.6
*PSMA6*	Proteasome subunit alpha type-6 (P60900)	23.4	47.27	2.53	8.56
*TPT1*	Translationally-controlled tumor protein (P13693)	26.04	64.58	13.01	35.49
*DSTN*	Destrin (P60981)	27.1	79.13	7.27	34.58
*TXN*	Thioredoxin (P10599)	34.2	88.24	209.2	431.8
*COPB2*	Coatomer subunit beta (P35606)	55.33	102.36	57.34	107.4
*STIP1*	Stress-induced-phosphoprotein 1 (P31948)	80.66	186.81	123	208.2
*PFN1*	Profilin-1 (P07737)	89.96	221.3	126.1	265.1
*NSF*	Vesicle-fusing ATPase (P46459)	93.46	206.55	41.33	76.3
*CFL1*	Cofilin-1 (P23528)	150.6	316.26	238.9	628.9

* LfQ (label free quantitation) are scores given * 10^8^; FTC-133 LfQ, proteins were derived from FTC-133 cells.

**Table 2:** Five exemplary proteins and their PTMs with location and short sequences before and after a modified site.

Gene Name	Modification	Location	Substrate Site
*RAB27B* (O00194)	*N*-acetylthreonine	2	MTDGDY
*XPOT* (O43592)	*N*-acetylmelthionine	1	MDEQA
	Glycyl lysine isopeptide (Lys-Gly) (interchain with G-Cter in ubiquitin)	557	KSLNKQMNP
	*N*6-acetyllysine	627	PLMEKFKIL
	Glycyl lysine isopeptide (Lys-Gly) (interchain with G-Cter in ubiquitin)	629	MEKFKILLE
	Glycyl lysine isopeptide (Lys-Gly) (interchain with G-Cter in ubiquitin)	634	ILLEKLMLA
	*N*6-acetyllysine	634	ILLEKLMLA
	Phosphothreonine	661	FASRTSKAF
*GM2A* (P17900)	*S*-cysteinyl 3-(oxidosulfanyl)alanine (Cys-Cys)	39	SWDNCDEGK
	*N*-linked (GlcNAc)	63	IVPGNVTLS
	*S*-cysteinyl 3-(oxidosulfanyl)alanine (Cys-Cys)	99	IKIPCTDYI
	*S*-cysteinyl 3-(oxidosulfanyl)alanine (Cys-Cys)	106	YIGSCTFEH
	*S*-cysteinyl 3-(oxidosulfanyl)alanine (Cys-Cys)	112	FEHFCDVLD
	*S*-cysteinyl 3-(oxidosulfanyl)alanine (Cys-Cys)	125	TGEPCPEPL
	*S*-cysteinyl 3-(oxidosulfanyl)alanine (Cys-Cys)	136	YGLPCHCPF
	*S*-cysteinyl 3-(oxidosulfanyl)alanine (Cys-Cys)	138	LPCHCPFKE
	*S*-cysteinyl 3-(oxidosulfanyl)alanine (Cys-Cys)	183	KRLGCIKIA
*THBS1* (P07996)	*N*-linked (GlcNAc)	248	NNVVNGSSP
	*C*-linked (Man)	385	GWSPWSEWT
	*O*-linked (Fuc)	394	SCSTSCGNG
	*O*-linked (GalNAc)	394	SCSTSCGNG
	*C*-linked (Man)	438	QDGGWSHWS
	*C*-linked (Man)	441	GWSHWSPWS
	*S*-cysteinyl 3-(oxidosulfanyl)alanine (Cys-Cys)	447	PWSSCSVTC
	*O*-linked (Fuc)	450	SCSVTCGDG
	*O*-linked (GalNAc)	450	SCSVTCGDG
	*S*-cysteinyl 3-(oxidosulfanyl)alanine (Cys-Cys)	451	CSVTCGDGV
	*S*-cysteinyl 3-(oxidosulfanyl)alanine (Cys-Cys)	462	RIRLCNSPS
	*S*-cysteinyl 3-(oxidosulfanyl)alanine (Cys-Cys)	474	NGKPCEGEA
	*S*-cysteinyl 3-(oxidosulfanyl)alanine (Cys-Cys)	484	ETKACKKDA
	*S*-cysteinyl 3-(oxidosulfanyl)alanine (Cys-Cys)	489	KKDACPING
	*C*-linked (Man)	498	GWGPWSPWD
	*O*-linked (Fuc)	507	ICSVTCGGG
	*N*-linked (GlcNAc)	1067	VKVVNSTTG
*PSMA1* (P25786)	*N*-acetylmethionine	1	MFRNQ
	Phosphoserine	14	VTVWSPQGR
	Glycyl lysine isopeptide (Lys-Gly) (interchain with G-Cter in ubiquitin)	30	MEAVKQGSA
	Glycyl lysine isopeptide (Lys-Gly) (interchain with G-Cter in ubiquitin)	39	YTVGLKSKTH
	Glycyl lysine isopeptide (Lys-Gly) (interchain with G-Cter in ubiquitin)	51	GLKSKTHAV
	Phosphoserine	54	KRAQSELAA
	Glycyl lysine isopeptide (Lys-Gly) (interchain with G-Cter in ubiquitin)	61	AAHQKKILH
	Glycyl lysine isopeptide (Lys-Gly) (interchain with G-Cter in ubiquitin)	115	LIGSKTQIP
	Glycyl lysine isopeptide (Lys-Gly) (interchain with G-Cter in ubiquitin)	208	DLTTKNVSI
	Phosphoserine	211	TKNVSIGIV
	Glycyl lysine isopeptide (Lys-Gly) (interchain with G-Cter in ubiquitin)	243	RPQRKAQPA
	Glycyl lysine isopeptide (Lys-Gly) (interchain with G-Cter in ubiquitin)	256	EPAEKADEP

## Supplementary Materials

The following are available online at http://www.mdpi.com/1422-0067/19/8/2257/s1.

## Abbreviations

µ*g*	Microgravity
PTM(s)	Posttranslational modification(s)
RPM	Random Positioning Machine
LfQ	label free quantitation
SKB	Semantic Knowledge Base
2D	Two-dimensional
3D	Three-dimensional
KE	Knowledge Explorer
RDF	Resource Description Framework
RPM	Random positioning machine
SPARQL	Semantic Protocol and RDF Query Language
